# MEK1/2 Inhibition Synergistically Enhances the Preventive Effects of Normobaric Oxygen on Spinal Cord Injury in Decompression Sickness Rats

**DOI:** 10.3389/fphys.2021.674430

**Published:** 2021-06-01

**Authors:** Quan Zhou, Xiangyang Meng, Guoyang Huang, Hongjie Yi, Juan Zheng, Kun Zhang, Weigang Xu

**Affiliations:** ^1^Department of Diving and Hyperbaric Medicine, Naval Special Medical Center, Naval Medical University, Shanghai, China; ^2^Department of Hyperbaric Oxygen, The First Affiliated Hospital, Naval Medical University, Shanghai, China

**Keywords:** decompression sickness, spinal cord injury, normobaric oxygen, MEK1/2, U0126, heat shock protein 32

## Abstract

A previous study from our team found that hyperbaric oxygen (HBO) pretreatment attenuated decompression sickness (DCS) spinal cord injury by upregulating heat shock protein 32 (HSP32) via the ROS/p38 MAPK pathway. Meanwhile, a MEK1/2-negative regulatory pathway was also activated to inhibit HSP32 overexpression. The purpose of this study was to determine if normobaric oxygen (NBO) might effectively induce HSP32 while concurrently inhibiting MEK1/2 and to observe any protective effects on spinal cord injury in DCS rats. The expression of HSP32 in spinal cord tissue was measured at 6, 12, 18, and 24 h following NBO and MEK1/2 inhibitor U0126 pretreatment. The peak time of HSP32 was observed at 12 h after simulated air diving. Subsequently, signs of DCS, hindlimb motor function, and spinal cord and serum injury biomarkers were recorded. NBO-U0126 pretreatment significantly decreased the incidence of DCS, improved motor function, and attenuated oxidative stress, inflammatory response, and apoptosis in both the spinal cord and serum. These results suggest that pretreatment with NBO and U0126 combined can effectively alleviate DCS spinal cord injury in rats by upregulating HSP32. This may lead to a more convenient approach for DCS injury control, using non-pressurized NBO instead of HBO.

## Introduction

Decompression sickness (DCS) is caused by inert gas bubbles forming in tissues and vessels following inadequate decompression in diving, aviation, and space activities (Vann et al., 2011). The overall rates of DCS per dive are 0.01–0.019% among recreational divers, 0.01–0.019% in scientific divers, 0.030% in U.S. Navy divers, and 0.095% in commercial divers (Vann et al., 2011; Mahon and Regis, 2014). Various tissues and organs are involved in DCS from the skin to the central nervous system, and the involvement of the spinal cord may cause serious sequelae, such as back pain, paresthesia, or acroparalysis (Blatteau et al., 2011; Saadi et al., 2019). Neurologic DCS accounts for 20–40% of all DCS, and spinal cord injury accounts for about 77% of neurologic DCS, even after active treatment, and more than 20% sequelae remain afterward (Blatteau et al., 2011; Mahon and Regis, 2014; Saadi et al., 2019). While complete resolution following treatment remains elusive, DCS, especially with spinal cord injury, is addressed mainly through prevention but, other than adhering to established decompression schedules, there are few other preventive strategies.

Hyperbaric oxygen (HBO), the practice of administering pure oxygen to patients while being exposed to ambient pressure greater than one absolute atmosphere, is widely used in DCS treatment (Moon, 2014). By increasing blood oxygen partial pressure and extending oxygen diffusion, HBO accelerates inert gas washout and attenuates hypoxic and ischemic damage caused by bubbles in tissues (Arieli et al., 2009). Numerous studies have shown that HBO also provides delayed protection by mobilizing intrinsic protective mechanisms against a variety of harms (Camporesi and Bosco, 2014; Gonzales-Portillo et al., 2019). Previous studies from our team showed that 280-kPa-60-min HBO pretreatment significantly reduced DCS incidence and alleviated DCS spinal cord injury in rats (Fan et al., 2010; Ni et al., 2013). Further research found that the neuroprotection of HBO was strongly associated with heat shock protein 32 (HSP32) upregulation and a signaling pathway involving ROS/p38 MAPK was discovered (Huang et al., 2014, 2016). Interestingly, while upregulating the expression of HSP32 in spinal cord neurons, HBO also activated the MEK1/2-negative regulatory pathway to prevent HSP32 overexpression (Huang et al., 2016). As an important effector of HBO, moderately expressed HSP32 exerts antioxidative, anti-inflammatory, and antiapoptotic effects, while overexpressed HSP32 results in numerous inimical effects (Schipper et al., 2019).

Although HBO is a safe, economical, and efficient therapy, its application in practice may at times be logistically limited by the need for a hyperbaric chamber, trained operators, and physicians, any of which may be unavailable at diving sites, or during high-altitude flights, submarine escape, or extra-vehicular activity in space. Since MEK1/2 works as a negative regulator, it is speculated that HSP32 can be induced effectively by a lower pressure of oxygen when the role of MEK1/2 is inhibited. The purpose of this study was to observe the effects of combined pretreatment with pure oxygen in a normobaric environment (normobaric oxygen, NBO) and MEK1/2 inhibition (inducted by specific inhibitor U0126) on spinal cord HSP32 expression and to record the beneficial effects on spinal cord injury in a rat DCS model.

## Materials and Methods

### Animals

A total of 194, 8-week-old adult male Sprague-Dawley (SD) rats weighing 300∼310 g were obtained from the Experimental Animal Center of the Naval Medical University (NMU). The animals were housed under a controlled light/dark cycle (12/12 h), temperature (24 ± 1°C), and relative humidity (54 ± 2%) and allowed to access a pelleted rodent diet and water *ad libitum* during the experiment.

### Procedure and Design

Part I. Rats were treated by intraperitoneal injection with U0126 (20 mg/kg, Selleck, United States) dissolved in 100 μL DMSO (Beyotime, China) 30 min before NBO exposure. The expression of HSP32 in rat spinal cord tissue was determined at 6, 12, 18, and 24 h following combined treatment with NBO and U0126. NBO and U0126 treatment were settled alone at the HSP32 peak time point of 12 h following NBO/Air exposure, as factorial design to reveal the interaction between NBO and U0126 in HSP32 induction. Six rats were investigated in each group, for measurement of the expression of HSP32.

Part II. Another 152 rats were randomly divided into five groups, 35 each for four simulated air dive groups and 12 for a control group. The four simulated air dive groups received treatments as follows: (1) NBO-U0126: treated with NBO and U0126; (2) NBO: treated with NBO only; (3) U0126: treated with U0126 and a sham exposure with normobaric air instead of NBO; and (4) Air: a sham exposure with normobaric air. Simulated air diving occurred at 12 h after either NBO and/or U0126 treatment. Following rapid decompression, the rats were observed for 30 min for DCS diagnosis followed by a 3-min motor function evaluation. Surviving rats were anesthetized by intraperitoneal injection with 3% pentobarbital sodium (1.5 ml/kg) 2 h after decompression. Blood and spinal cord tissues were sampled for biochemical analysis. Control rats were similarly sampled without any treatments or simulated air diving.

**FIGURE 1:**
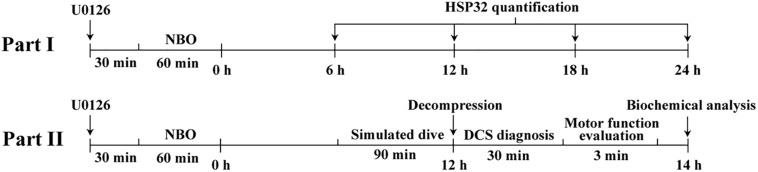
The timeline and design of the study. Part I, NBO-U0126 treatment-induced HSP32 expression in rat spinal cord was determined at 6, 12, 18, and 24 h following NBO exposure. The separate NBO and U0126 treatment HSP32 samples were collected at 12 h following NBO exposure. Part II, rats in different treatment groups underwent simulated air dives, concluding that at the HSP32 peak time point of 12 h, DCS diagnosis, motor function evaluation, and sample collection for biochemical analysis were successively carried out.

Figure 1 shows the timeline of the experiments.

### NBO Exposure

A 100-kPa-60-min NBO exposure was performed with rats inside a transparent hyperbaric rodent chamber (RDC 150-300-6, NMU, China). The rats were placed in the chamber, and it was flushed with pure oxygen for 5 min to replace the air inside, then it was continuously ventilated with pure oxygen for 60 min. The Air group and U0126 group were similarly exposed but with normobaric air instead of NBO.

### Simulated Air Dive

Rats in four simulated air dive groups were compressed with air in a hyperbaric chamber (RDC 150-300-6) 10.5 h after either NBO-U0126 pretreatment or NBO/U0126/Air treatment, one rat at a time from each group. Compression to 700 kPa took 5 min and was maintained for 90 min. Decompression was linear to ambient pressure at 200 kPa/min in 3 min. During exposure, the chamber was continuously ventilated with air to minimize any decrease of oxygen and accumulation of carbon dioxide.

### DCS Diagnosis

To standardize the activity level and facilitate DCS diagnosis, rats were subjected for 30 min to walk inside an electrically controlled cylindrical cage rotating at a perimeter speed of 3 m/min. During this time, two observers who were blinded to the treatments diagnosed DCS. According to previous studies from our team, DCS diagnosis was based on observation of any of the following symptoms: respiratory distress, walking difficulties, limb paralysis, rolling with cage rotation, convulsions, or death (Fan et al., 2010; Ni et al., 2013; Zhang et al., 2015, 2016, 2017; Yu et al., 2020). A binary classification of “no-DCS” and “DCS” was recorded; whenever any symptoms described above were observed, the latency was also recorded.

### Motor Function Evaluation

Immediately after a DCS diagnosis, the Basso–Beattie–Bresnahan (BBB) scale was used to evaluate the motor function of surviving rats. The score ranged from 0 to 21, with 0 indicating complete hindlimb disability and 21 indicating normal motor function; these scores are defined by the combinations of joint and limb movements, limb coordination, trunk stability, steps, paw placement, and tail position (Bhimani et al., 2017). Rats were allowed to walk freely for 3 min in an open field. Hindlimb movements and coordination were observed and scored by two experienced observers who were blinded to the treatments. All rats displayed normal motor function before the experiment.

### HSP32 Quantification

Western blot analysis was performed to quantify spinal cord HSP32. In brief, 2 h after simulated air diving rats were euthanized by anesthesia overdose, then T10-L4 thoracolumbar spinal cords were removed and homogenated. Protein samples were collected and concentration determined with BCA Kits (Beyotime). The samples were electrophoresed and transferred to polyvinylidene fluoride membranes. Membranes were incubated with rabbit monoclonal primary antibodies directed against rat HSP32 (ab13243, Abcam, United Kingdom) and β-actin (4970, CST, United States). Proteins were visualized using infrared anti-rabbit IgG (5151, CST), and the intensity of each band was imaged in an Odyssey imaging system (Li-Cor Bioscience, United States). Ratios of HSP32 to β-actin were calculated.

### Biochemical Analysis

Spinal cords were sampled in surviving rats 2 h after simulated air diving as described above. Venous blood was drawn from the postcava at the same time, then serum was collected after centrifuging. Spinal cord and serum levels of interleukin-1β (IL-1β), tumor necrosis factor-α (TNF-α), and neuron-specific enolase (NSE) were assayed by ELISA kits (Jiancheng Bioengineering Institute, China). Malondialdehyde (MDA) was detected by thiobarbituric acid colorimetric methods assay kits (Beyotime). Caspase-3 activity was determined by spectrophotometry assay kits (Jiancheng). All assays were performed following the respective manufacturer’s instructions.

### Histological Examination

T10-L4 thoracolumbar spinal cords were postfixed in 4% formaldehyde, embedded in paraffin, sectioned serially, and mounted on silane-covered slides. Immunohistochemical staining was performed in two rounds: firstly, using the Neun antibody (ab177487, Abcam) and TYR-488 regent (Ex/Em: 490/520 nm, Shanghai Rainbow, China), and secondly, performing Tunel staining using the Tunel-CY3 kit (Ex/Em: 550/570 nm, Shanghai Rainbow) with standard protocols. Antigen retrieval was preformed between each step. Finally, nuclei were labeled by DAPI (Ex/Em: 358/461 nm, Shanghai Rainbow), sections were examined at a magnification of ×400, and three fields were randomly chosen to determine Tunel-positive cells by using a computer image analysis system (DS-U3, NIKON, Japan).

### Statistical Analysis

Preliminary experiments indicated that 73% vs. 40% of DCS incidence should be expected for Air and NBO-U0126 treatments, respectively, and 35 rats per group were estimated as adequate for 80% power to show a difference in the incidence of DCS, based on a two-tailed significance level of 0.05. All data are presented as mean ± SD, except mortality, DCS incidence, and BBB score. A chi-square test was conducted to compare mortality or DCS incidence between different groups. BBB scores were analyzed using a Mann–Whitney test. Continuous variables were tested for normal distribution with the Kolmogorov–Smirnov test and compared among groups by one-way ANOVA via a Student–Newman–Keuls test or *post hoc* Dunnett’s test. The threshold for significance was accepted at *P* < 0.05. SPSS 22.0 (SPSS Inc., United States) was used for statistical analysis.

## Results

### Effects of NBO-U0126 on HSP32 Expression in Rat Spinal Cord

**FIGURE 2:**
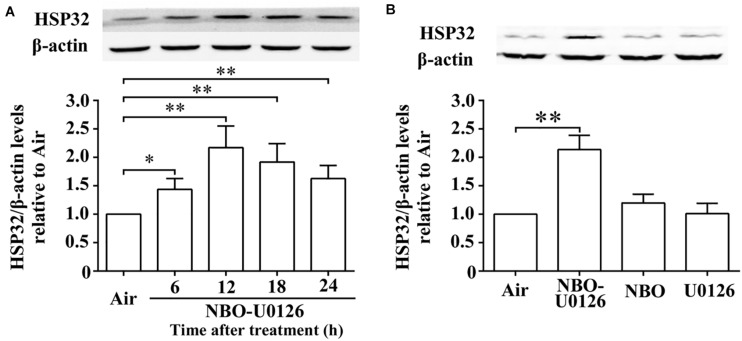
Effects of NBO-U0126 on the expression of HSP32 in rat spinal cord. Rats were exposed with NBO and/or U0126, and spinal cord HSP32 was determined at designated times following treatment. **(A)** Time course expression of HSP32 (*n* = 6). **(B)** HSP32 expression at 12 h with different treatments (*n* = 6, the original image see in Supplementary Material). ^∗^*P* < 0.05, ^∗∗^*P* < 0.01.

Western blot results showed that the expression of HSP32 in rat spinal cords increased significantly after NBO-U0126 treatment and peaked at 12 h (*P* < 0.001, Figure 2A). The factorial design analysis showed that neither NBO nor U0126 alone impacted on HSP32 expression (*P* > 0.05), but NBO and U0126 had a positive interaction on HSP32 upregulation in the NBO-U0126 treatment group (*P* < 0.001, Figure 2B).

### Effects of NBO-U0126 on the Incidence and Mortality of DCS

**FIGURE 3:**
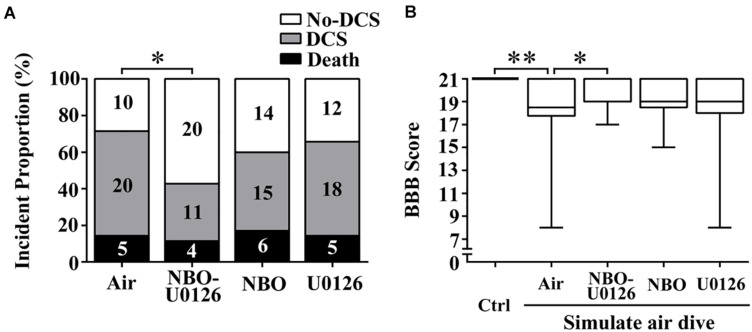
Effects of NBO-U0126 on DCS incidence and motor function in rats. Rats were treated with NBO and/or U0126, 12 h prior to simulated air dives. The BBB scale was used to evaluate rat hindlimb motor function. **(A)** Incidence of DCS (*n* = 35, the numeric values in the bars are numbers of rats); **(B)** motor function scores in all surviving rats (Ctrl n = 12, Air *n* = 30, NBO-U0126 *n* = 31, NBO *n* = 29, U0126 *n* = 30). ^∗^*P* < 0.05, ^∗∗^*P* < 0.01.

The incidence of DCS in the NBO-U0126 group was significantly lower than in the Air group (*P* = 0.029, Figure 3A). Neither NBO nor U0126 alone had a significant impact on DCS incidence (*P* > 0.05), and no treatment made a significant difference to the latency or proportion of mortality in rats (*P* > 0.05).

### Effects of NBO-U0126 on Motor Function and Spinal Cord Apoptosis After a Simulated Air Dive

**FIGURE 4:**
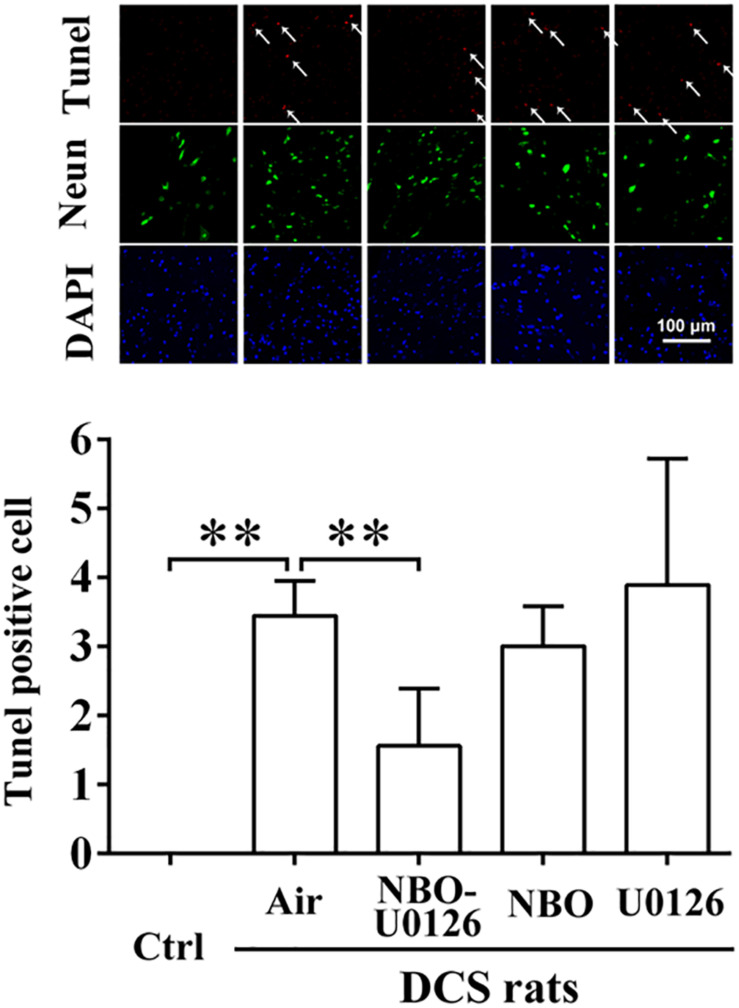
Effect of NBO-U0126 on spinal cord apoptosis in DCS rats. Thoracolumbar spinal cords were sampled from surviving DCS rats at 2 h after simulated air dives, for Tunel/Neun immunohistochemistry to reveal cell apoptosis. Tunel-positive cells are labeled by arrows. ^∗∗^*P* < 0.01. (*n* = 6).

BBB scores were markedly reduced after simulated diving, compared with controls (*P* < 0.001, Figure 3B). NBO-U0126 significantly increased the impaired scores in surviving rats (*P* = 0.035, Figure 3B). Tunel-positive cell numbers were markedly increased in DCS rats (*P* < 0.001, Figure 4), and NBO-U0126 reduced the impairment (*P* = 0.001, Figure 4).

### Effects of NBO-U0126 on Spinal Cord and Serum Biomarkers After a Simulated Air Dive

**FIGURE 5:**
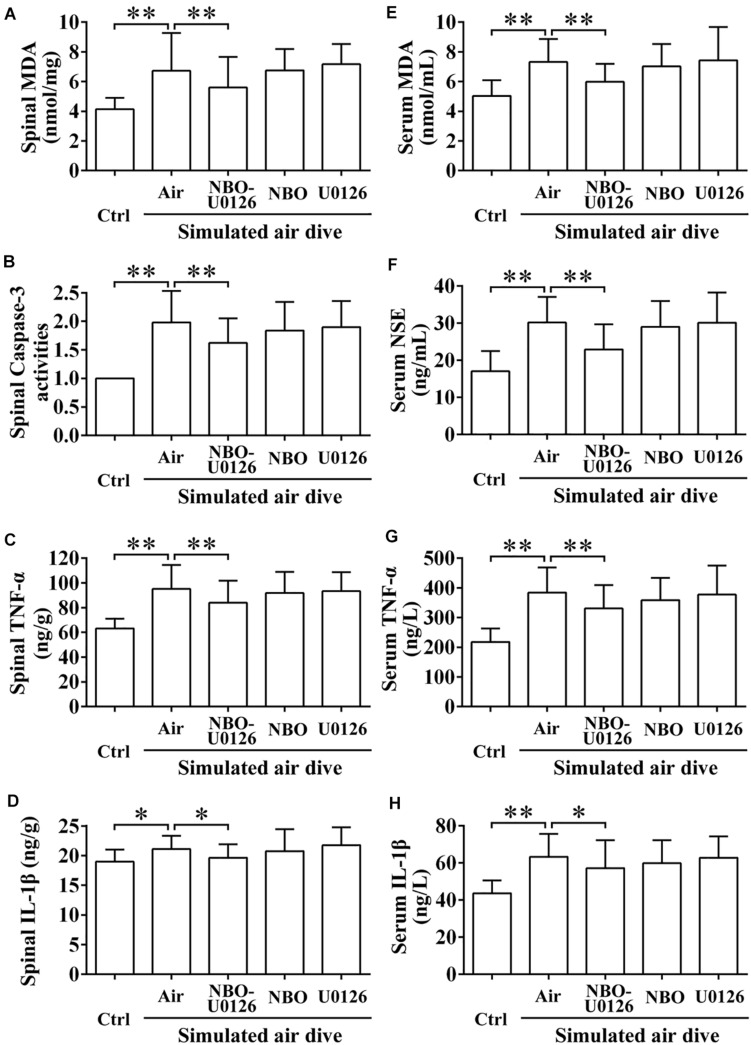
Effects of NBO-U0126 on spinal cord and serum biomarkers after simulated air dive. Spinal cord and serum biomarkers were measured in all surviving rats 2 h after simulated air dive (Ctrl *n* = 12, Air *n* = 30, NBO-U0126 *n* = 31, NBO *n* = 29, U0126 *n* = 30). **(A–D)** Spinal cord biomarkers; **(E–H)** Serum biomarkers. ^∗^*P* < 0.05, ^∗∗^*P* < 0.01.

After simulated air diving, all the spinal cord and serum injury biomarkers increased significantly (*P* < 0.05, Figure 5). NBO-U0126 reduced the deterioration of these parameters in surviving rats (*P* < 0.05, Figure 5).

## Discussion

DCS spinal cord injury is a critical medical concern in hyper-/hypo-baric exposures, which usually comes with severe symptoms and sequelae, and the pathogenesis is not fully clarified yet (Francis, 1998). Studies have found that decompression bubbles could induce spinal ischemia and inflammatory and oxidative stress, which may play important parts in the pathogenesis of DCS spinal cord injury (Hallenbeck et al., 1975; Francis, 1998). HSP32, also named heme oxygenase-1 (HO-1), has antioxidative, anti-inflammatory, and antiapoptotic properties by decomposing free heme and producing protective products including Fe^2+^, carbon monoxide, and biliverdin (Gozzelino et al., 2010). Upregulation of HSP32 has been confirmed to alleviate a variety of central nervous system injuries including trauma, stroke, tumor, and neurodegeneration, especially in the spinal cord (Gozzelino et al., 2010; Sferrazzo et al., 2020).

Previous research from our team showed that a single exposure to 280 kPa HBO for 60 min reduced DCS spinal cord injury in rats (Ni et al., 2013), and ROS/p38 MAPK-mediated HSP32 upregulation was found to participate the process (Huang et al., 2014, 2016). Interestingly, a MEK1/2-mediated negative regulatory pathway was found to be simultaneously involved, and inhibiting MEK1/2 led to overexpression of HSP32 (Huang et al., 2016). The results suggested that the 280-kPa-60-min HBO exposure may be excessive just for HSP32 expression, and a more moderate exposure regimen may be efficient in inducing HSP32 if the negative regulation is removed. The results in the present study verified this hypothesis, showing that the combined administration of NBO and MEK1/2 inhibitor U0126 achieved similar effects upon HSP32 expression as HBO did (Huang et al., 2014). Furthermore, NBO treatment alone showed only a non-significant slight increase in HSP32 expression, while U0126 alone had no effect.

Further observation identified that combined NBO-U0126 pretreatment markedly decreased the incidence of DCS from 71 to 43% in rats, which was not a simple overlay but a significant synergistic effect of the two treatments combined. The beneficial effect of NBO-U0126 upon DCS incidence was similar to that of the 280-kPa HBO treatment found in a previous study from our team (from 67 to 37%) (Ni et al., 2013).

Due to the abundance of lipids and lack of collateral circulation, the spinal cord is more prone to bubble damage (Francis, 1998; Huang et al., 2014). The current study again observed obvious signs and biomarker changes of spinal cord injury in all the simulated diving rats except those treated with NBO-U0126, in which group most of the deteriorated parameters were alleviated. Such effects were not observed among rats treated with NBO or U0126 alone. This appeared to be correlated with HSP32 expression, for neither NBO nor U0126 alone improved HSP32 expression in the spinal cord. By decomposing oxidative free heme and producing reductive biliverdin and Fe^2+^, HSP32 enables antioxidation (Gozzelino, 2016). Carbon monoxide, another metabolin of HSP32, is able to mediate anti-apoptosis and anti-inflammation via the sGC pathway (Ryter, 2019). While oxidative stress, inflammation, and apoptosis are pivotal pathogenesis in DCS spinal cord injury, the beneficial outcomes from the NBO-U0126-treated rats suggest that HSP32 participates in the process of DCS spinal cord injury.

NBO-U0126 pretreatment had limited impact on the mortality in rats in the current study. This may be related to the rapid formation of bubbles and subsequent embolism after decompression, leading to acute cardiopulmonary failure (Yount, 1979; Wisløff and Brubakk, 2001). Bubbles are likely the pathogenic factor for DCS, but in a previous study from our team, reducing bubble formation did not improve in the preventive effect of HBO pretreatment in rats (Ni et al., 2013). All mortal cases in this study occurred suddenly with respiratory distress or convulsions in seconds and were consistent with acute circulatory embolism (Yount, 1979; Wisløff and Brubakk, 2001).

Given the systemic effects of oxygen breathing and intraperitoneal administration of U0126, the protection of NBO-U0126 pretreatment upon DCS is possibly not limited to the spinal cord. The improvement of serum oxidative and inflammatory markers may be an indication of systemic protection. Although such improvements related to the alleviation of DCS spinal cord injury, the improvement of NSE may indicate reduced damage to the nervous system. Among the parameters investigated, the increase and improvement of IL-1β were more pronounced in serum than in spinal cord tissue. Given that serum IL-1β was reported responsible for circulating microparticle-mediated DCS vascular injuries (Thom et al., 2018), our results support a potential systemic effect of NBO-U0126 pretreatment.

In summary, pretreatment with NBO and MEK1/2 inhibitor U0126 combined efficiently reduced the incidence of DCS and alleviated DCS spinal cord injury via the antioxidative, anti-inflammatory, and anti-apoptotic effects by HSP32 upregulation in the spinal cord. To our knowledge, this may be the first report that atmospheric pure oxygen can reduce DCS injury severity with greater effect using the synergism of a chemical agent. To fully realize the beneficial effects of hyperbaric treatment under atmospheric pressure, where a hyperbaric chamber is not available, the application of normobaric oxygen administration could be augmented in support of various hyper-/hypo-baric practices. Further research on the mechanisms underpinning these results is under way.

## Data Availability Statement

The raw data supporting the conclusions of this article will be made available by the authors, without undue reservation.

## Ethics Statement

The animal study was reviewed and approved by the Ethics Committee for Animal Experiments of the Naval Medical University.

## Author Contributions

WX, GH, and QZ designed the experiments. QZ, XM, HY, and GH conducted the experiments. QZ, XM, JZ, and GH contributed to data analyses and interpretation of the results. QZ, XM, GH, KZ, and WX wrote the manuscript and prepared all the figures. All authors contributed to the article and approved the submitted version.

## Conflict of Interest

The authors declare that the research was conducted in the absence of any commercial or financial relationships that could be construed as a potential conflict of interest.
