# Supplementary material for: MEK1/2 Inhibition Synergistically Enhances the Preventive Effects of Normobaric Oxygen on Spinal Cord Injury in Decompression Sickness Rats
Source: Front Physiol. 2021 Jun 1;12:674430. doi: 10.3389/fphys.2021.674430 (PMC8204088; doi:10.3389/fphys.2021.674430)
Supplement: Supplementary file 1 [file Image_1.PDF]

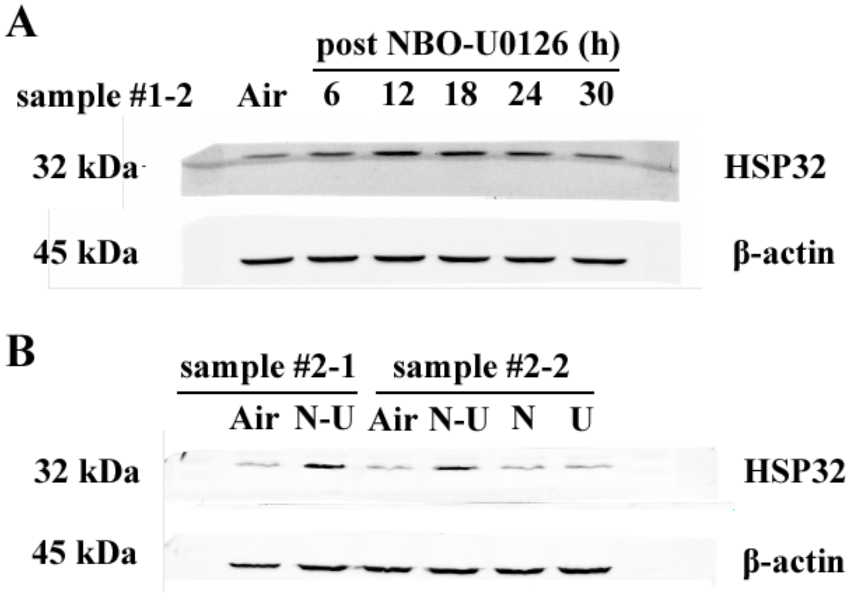

Fig.1 The original image from western blot gel. Western blot analysis was performed to quantify spinal cord HSP32 as describes in Material and Methods. Membranes were cut for primary antibodies incubation. (A) Time course expression of HSP32; (B) HSP32 expression at 12 h with different treatments. N-U: NBO-U0126, N: NBO, U: U0126.
